# HIV infection increases the risk of acquiring *Plasmodium vivax* malaria: a 4-year cohort study in the Brazilian Amazon HIV and risk of vivax malaria

**DOI:** 10.1038/s41598-022-13256-4

**Published:** 2022-05-31

**Authors:** Cecilia Victoria Caraballo Guerra, Bernardo Maia da Silva, Pia Müller, Djane Clarys Baia-da-Silva, Marco Antônio Saboia Moura, José Deney Alves Araújo, Juan Carlo Santos e Silva, Alexandre Vilhena Silva-Neto, Antonio Alcirley da Silva Balieiro, André Guilherme da Costa-Martins, Gisely Cardoso Melo, Fernando Val, Quique Bassat, Helder I. Nakaya, Flor Ernestina Martinez-Espinosa, Marcus Lacerda, Vanderson Souza Sampaio, Wuelton Monteiro

**Affiliations:** 1 Universidade Do Estado Do Amazonas, Manaus, Amazonas Brasil; 2 Fundação de Medicina Tropical Dr. Heitor Vieira Dourado, Av. Pedro Teixeira, 25, Dom Pedro, Manaus, Amazonas 69040-000 Brazil; 3 London School of Hygiene and Tropical Medicine, London, England; 4 Instituto Leônidas and Maria Deane, Fundação Oswaldo Cruz, Manaus, Amazonas Brazil; 5 USP Centro de Inovação, Universidade de São Paulo, Sao Paulo, Brazil; 6 Departamento de Análises Clínicas E Toxicológicas, Faculdade de Ciências Farmacêuticas, Universidade de São Paulo, Sao Paulo, Brazil; 7 Plataforma Científica Pasteur-USP, Universidade de São Paulo, Sao Paulo, Brazil; 8 ISGlobal, Hospital Clínic, Universitat de Barcelona, Barcelona, Spain; 9 Centro de Investigação Em Saúde de Manhiça (CISM), Maputo, Mozambique; 10 ICREA, pág. Lluís Companys 23, 08010 Barcelona, Spain; 11 Departamento de Pediatria, Hospital Sant Joan de Déu, Universitat de Barcelona, Esplugues, Barcelona, Spain; 12 Consorcio de Investigación Biomédica en Red de Epidemiología y Salud Pública (CIBERESP), Madrid, Spain; 13 Hospital Israelita Albert Einstein, Sao Paulo, Brazil

**Keywords:** HIV infections, Malaria

## Abstract

Globally, malaria and human immunodeficiency virus (HIV) are both independently associated with a massive burden of disease and death. While their co-infection has been well studied for *Plasmodium falciparum*, scarce data exist regarding the association of *P. vivax* and HIV. In this cohort study, we assessed the effect of HIV on the risk of vivax malaria infection and recurrence during a 4-year follow-up period in an endemic area of the Brazilian Amazon. For the purpose of this study, we obtained clinical information from January 2012 to December 2016 from two databases. HIV screening data were acquired from the clinical information system at the tropical hospital *Fundação de Medicina Tropical Dr. Heitor Vieira Dourado* (FMT-HVD). The National Malaria Surveillance database (SIVEP malaria) was utilized to identify malaria infections during a 4-year follow-up period after diagnosis of HIV. Both datasets were combined via data linkage. Between 2012 and 2016, a total of 42,121 people were screened for HIV, with 1569 testing positive (3.7%). Out of all the patients diagnosed with HIV, 198 had at least one episode of *P. vivax* malaria in the follow-up. In the HIV-negative group, 711 participants had at least one *P. vivax* malaria episode. When comparing both groups, HIV patients had a 6.48 [(5.37–7.83); *P* < 0.0001] (adjusted relative risk) greater chance of acquiring *P. vivax* malaria. Moreover, being of the male gender [ARR = 1.41 (1.17–1.71); *P* < 0.0001], Amerindian ethnicity [ARR = 2.77 (1.46–5.28); *P* < 0.0001], and a resident in a municipality of the Metropolitan region of Manaus [ARR = 1.48 (1.02–2.15); *P* = 0.038] were independent risk factors associated with an increased risk of clinical malaria. Education ≥ 8 years [ARR = 0.41 (0.26–0.64); *P* < 0.0001] and living in the urban area [ARR = 0.44 (0.24–0.80); *P* = 0.007] were associated to a lower risk of *P. vivax* malaria. A total of 28 (14.1%) and 180 (25.3%) recurrences (at least a second clinical malaria episode) were reported in the HIV-positive and HIV-negative groups, respectively. After adjusting for sex and education, HIV-positive status was associated with a tendency towards protection from *P. vivax* malaria recurrences [ARR = 0.55 (0.27–1.10); *P* = 0.090]. HIV status was not associated with hospitalizations due to *P. vivax* malaria. CD4 + counts and viral load were not associated with recurrences of *P. vivax* malaria. No significant differences were found in the distribution of parasitemia between HIV-negative and HIV-positive *P. vivax* malaria patients. Our results suggest that HIV-positive status is a risk factor for vivax malaria infection, which represents an additional challenge that should be addressed during elimination efforts.

## Introduction

Malaria and HIV/AIDS are major public health problems and globally have a great geographical overlap, with most affected people living in sub-Saharan Africa, the Indian subcontinent and Southeast Asia, Latin America, and the Caribbean^1^. This overlap favors *Plasmodium*-HIV co-infections, which are responsible for deaths of millions of individuals each year^2–4^. Although the consequences of co-infection with HIV-*Plasmodium* are not fully understood, the available evidence suggests that infections act synergistically and together may result in worse clinical outcomes^3–5^. Recent studies show that people living with HIV have more episodes of severe and lethal *P. falciparum* malaria, and more malaria treatment failures^3–9^. HIV can increase parasitic loads in malaria patients and consequently increase malaria transmission rates^7,10–12^. Moreover, *P. falciparum* infection may increase HIV plasma viral load and reduce TCD4 + cells^13,14^; this, in turn, suggests that malaria can lead to a faster progression from HIV infection to AIDS and increased risk of therapeutic failure after antimalarial chemotherapy^15^. However, the evidence on HIV-*Plasmodium* co-infection is mostly restricted to *P. falciparum* endemic areas in sub-Saharan Africa where the two diseases have the highest burden^16^.

The impact of HIV on susceptibility to non-*P. falciparum* malaria is unknown, and further research should be conducted in other settings to assess the interaction of HIV infection with other *Plasmodium* species, such as *P. vivax*^17^. Globally, ~ 229 million malaria cases were reported in 2019, with at least 6.9 million cases due to *P. vivax* (3%)^18^. Despite its comparatively lower numbers, *P. vivax* is geographically the most widely distributed cause of malaria, with its highest endemicity found in Southeast Asia, Central Asia, and Latin America, and a much less frequent presence in Africa^19^. *P. vivax* is a challenging parasite to eliminate due to its peculiar biology, which causes the different origins of a *P. vivax* malaria episode that include acquired infection by mosquito bites, relapse from liver-stage hypnozoites, and recrudescence from blood-stage treatment failure^20^. Clinically, *P. vivax* infection ranges from asymptomatic individuals carrying low parasitemia to severe disease and death^21^. Reports of HIV-*P. vivax* co-infection are scarce in the literature, with only a few studies describing their clinical outcomes^22–27^. In one study in the Brazilian Amazon, of 21 patients with HIV-*P. vivax* co-infection hospitalized in a referral hospital, severe malaria was diagnosed in 5 (23.8%), and one patient died (4.8%)^27^.

Although over the past three decades, there has been clear evidence of an association between the increased risk of *falciparum* malaria among HIV-infected patients, research on co-infection with the most widely distributed *vivax* malaria has so far been neglected. In this study, we evaluated the impact of HIV infection on *P. vivax* malaria incidence and the frequency of recurrences in a malaria endemic area of the Brazilian Amazon. The hypothesis of this study was that HIV infection may increase *P. vivax* malaria susceptibility and recurrence rates.

## Results

### Participant characteristics

**Figure 1:**
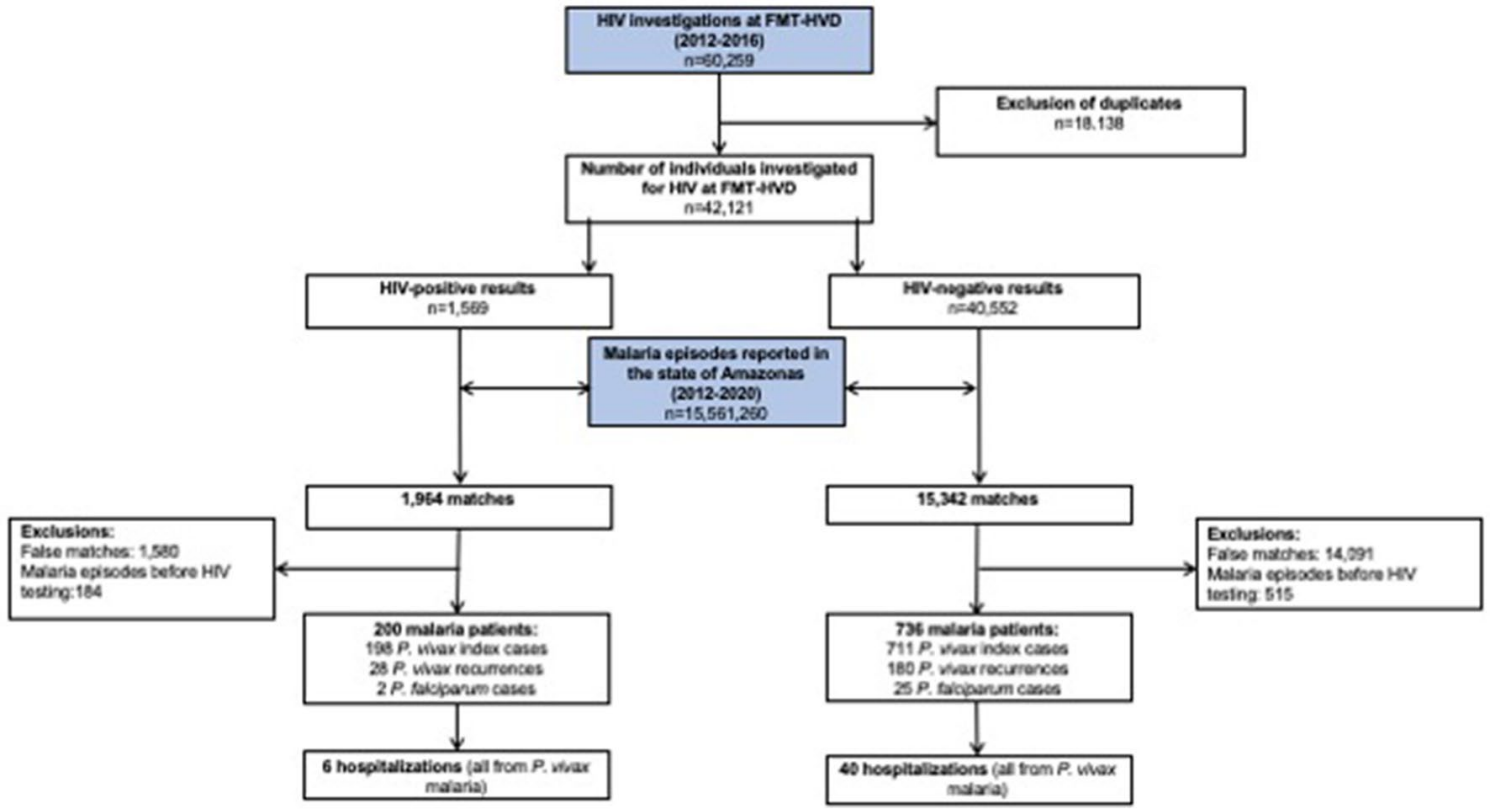
Flowchart of participants’ inclusion and linkage between HIV status and malaria episode databases, Amazonas state, Brazil.

A total of 60,259 HIV tests were performed at the *Fundação de Medicina Tropical Dr. Heitor Vieira Dourado* (FMT-HVD) between January 2012 and December 2016. After removing duplicates for individuals tested more than once, 42,121 were included in the study, with 1569 being classified as HIV- positive (3.7%) and 40,552 as HIV-negative (96.3%) (Fig. 1). Compared with the HIV-negative group, the HIV-positive group had a higher proportion of males (*P* < 0.001) and a younger mean age (*P* < 0.001). Regarding municipality of residence, HIV-positive status was less prevalent in Manaus compared to the municipalities of the metropolitan region (*P* = 0.046) (Table 1).

**Table 1:** Demographic characteristics of the included participants according to HIV status, tested at the FMT-HVD, Brazil, between 2012 and 2016.

Variable	HIV status			P-value
	Total (n = 42,121)	Negative (40,552)	Positive (1569)	
Male	23,110/42,121 (54.9%)	22,046/40,552 (54.4%)	1064/1569 (67.8%)	< 0.001
Mean age (years; ± SD)	37.7 ± 15.5	37.7 ± 15.5	35.7 ± 14.2	< 0.001
Education (years)				0.036
< 3#	385/20,410 (2%)	361/19,341 (2%)	24/1069 (2%)	
4–7	7702/20,410 (37.7%)	7263/19,341 (37.5%)	439/1069 (41%)	
≥ 8	12,323/20,410 (60.3%)	11,717/19,341(60.5%)	606/1069 (57%)	
Ethnicity (self-declared)				0.43
Admixed#	21,906/24,395(89.8%)	20,818/23,194(89.8%)	1088/1201 (90.6%)	
White	1918/24,395(7.9%)	1832/23,194 (7.9%)	86/1201 (7.2%)	
Black	421/24,395(1.7%)	398/23,194 (1.7%)	23/1201 (1.9%)	
Amerindian	150/24,395(0.6%)	146/23,194(0.6%)	4/1201 (0.3%)	
Place of residence				0.20
Urban área	40,847/40,983 (99.7%)	39,298/39,426 (99.7%)	1549/1557 (99.5%)	
Rural área	136/40,983 (0.3%)	128/39,426 (0.3%)	8/1557 (0.5%)	
Residence municipality				0.064
Manaus#	38,358/41,021 (93.5%)	36,924/39,464 (93.6%)	1434/1557 (92.1%)	
Metropolitan region	1381/41,021 (3.4%)	1315/39,464 (3.3%)	66/1557 (4.2%)	
Others	1282/41,021 (3.1%)	1225/39,464 (3.1%)	57/1557 (3.7%)	

^#^Reference groups.

### HIV-status and malaria episode database matches

A total of 15,561,260 malaria tests were performed in the state of Amazonas between January 2012 and December 2020, according to the SIVEP-malaria database. The linkage process between the databases for malaria and HIV resulted in 1,954 and 15,342 matches between HIV-positive and HIV-negative groups, respectively. After a detailed analysis and further exclusion of false matches and malaria episodes reported before HIV testing, a total of 936 participants were identified as having had at least one episode of *P. vivax* malaria during follow-up, 198 in the HIV-positive group and 711 in the HIV-negative group (Fig. 1).

### Impact of HIV status and other variables on malaria incidence

Table 2 summarizes the results of the univariate and multivariate logistic regression models and evaluates the factors associated with *P. vivax* malaria incidence. HIV-positive status [ARR = 6.48 (5.37–7.83); *P* < 0.0001], male gender [ARR = 1.41 (1.17–1.71); *P* < 0.0001], Amerindian ethnicity [ARR = 2.77 (1.46–5.28); *P* = 0.002], and residence in a municipality in the Metropolitan region of Manaus [ARR = 1.48 (1.02–2.15); *P* = 0.038] were independently associated with an increased risk of *P. vivax* malaria. Education ≥ 8 years [ARR = 0.41 (0.26–0.64); *P* < 0.0001] and living in the urban area [ARR = 0.44 (0.24–0.80); *P* = 0.007] were independently associated to a decreased risk of *P. vivax* malaria.

**Table 2:** Impact of HIV-status and other variables on *P. vivax* malaria incidence in the state of Amazonas, Brazil, 2012–2020.

Variable	RR (CI95%)	*P*	ARR (CI95%)	*P*
HIV-positive	7.20 (6.20–8.36)	< 0.0001	6.48 (5.37–7.83)	< 0.0001
Male	1.59 (1.39–1.82)	< 0.0001	1.41 (1.17–1.71)	< 0.0001
Age (years; ± SD)	1.00 (0.99–1.01)	0.412		
Education (years)				
< 3#				
4–7	0.68 (0.43–1.06)	0.092	0.75 (0.48–1.16)	0.192
≥ 8	0.34 (0.22–0.54)	< 0.0001	0.41 (0.26–0.64)	< 0.0001
Ethnicity				
Admixed#				
White	0.80 (0.36–1.77)	0.577		
Black	1.17(0.85–1.61)	0.336		
Amerindian	4.47 (2.49–8.04)	< 0.0001	2.77 (1.46–5.28)	0.002
Place of residence				
Urban area				
Rural area	0.23 (0.13–0.38)	< 0.0001	0.44 (0.24–0.80)	0.007
Residence municipality				
Manaus#				
Metropolitan region	1.65 (1.24–2.20)	0.001	1.48 (1.02–2.15)	0.038
Others	1.59 (1.18–2.16)	0.002	1.36 (0.91–2.03)	0.135

^#^Reference groups.

### Impact of HIV status on recurrences of *P. vivax* malaria

**Figure 2:**
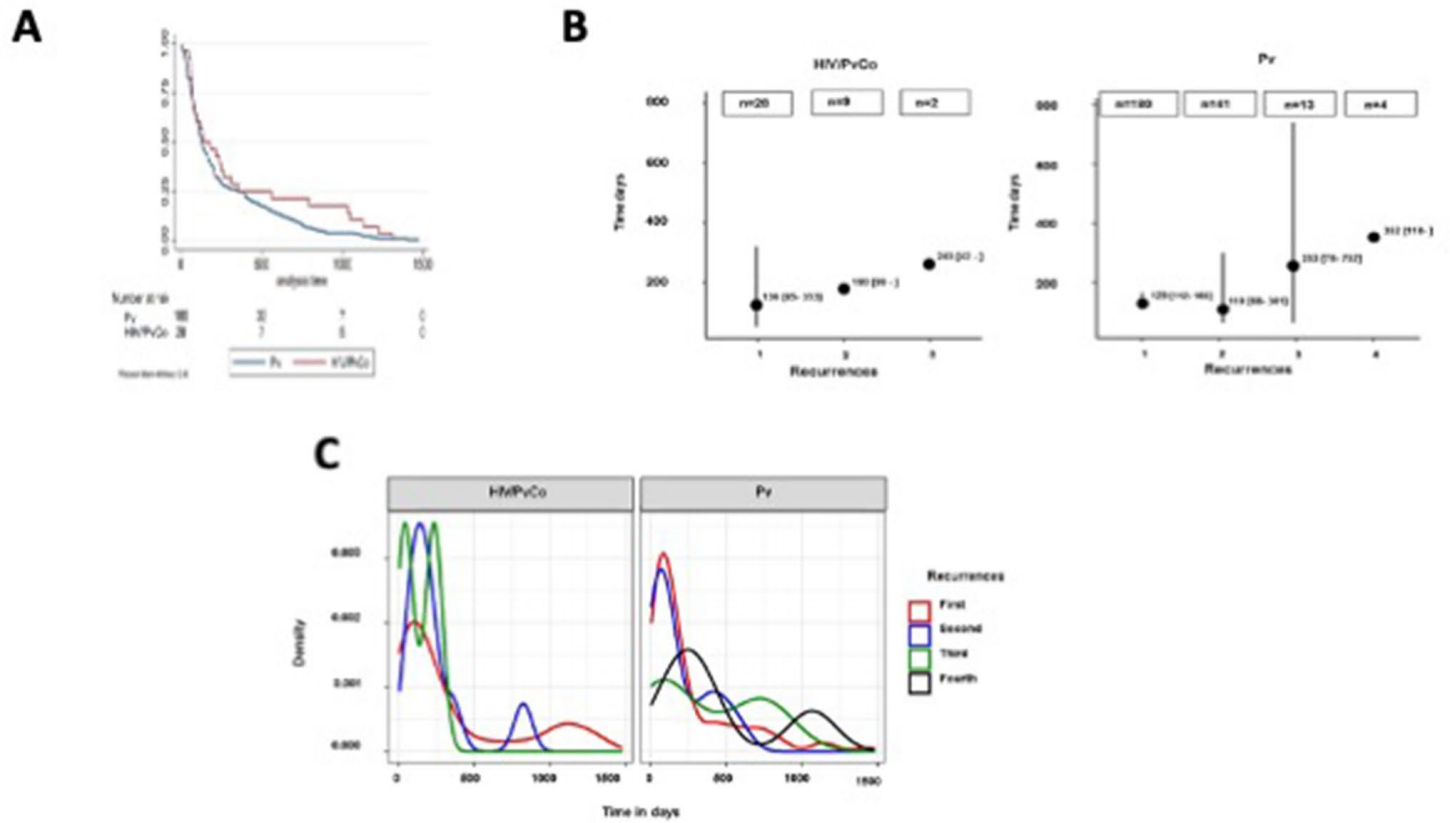
(**A**) Kaplan–Meier survival estimates comparing the time to first *Plasmodium vivax* malaria recurrence (in days). (**B**) Median of the number of *Plasmodium vivax* malaria recurrences by HIV status groups throughout the follow-up. (**C**) Graphical representation of *Plasmodium vivax* malaria recurrences densities throughout the follow-up. Time zero was the date of the index case for all recurrences.

A total of 28 (14.1%) and 180 (25.3%) recurrences were reported in the HIV-positive and HIV-negative groups, respectively. In the univariate analysis, HIV-positive status was associated with protection from recurrences of *P. vivax* malaria [RR = 0.57 (0.40–0.82); *P* = 0.003]. After adjusting for sex and education, the association between HIV-positive status with protection from recurrences was not detected [ARR = 0.55 (0.27–1.10); *P* = 0.090] (Table 3 and Fig. 2).

**Table 3:** Impact of HIV-status and other variables on *P. vivax* recurrences in the state of Amazonas, Brazil, 2012–2020.

Variable	RR (CI95%)	*P*	ARR (CI95%)	*P*
HIV-positive	0.57 (0.40–0.82)	0.003	0.55 (0.27–1.10)	0.090
Male	0.80 (0.63–1.03)	0.078	0.75 (0.47–1.19)	0.215
Age (years; ± SD)	1.00 (0.99–1.01)	0.935		
Education (years)				
< 3#				
4–7	0.63 (0.31–1.27)	0.197	1.06 (0.29–3.88)	0.928
≥ 8	0.59 (0.30–1.17)	0.131	1.06 (0.30–3.77)	0.922
Ethnicity				
Admixed#				
White	0.52 (0.08–3.41)	0.495		
Black	0.72 (0.42–1.22)	0.221		
Amerindian	1.04 (0.39–2.74)	0.938		
Place of residence				
Urban area				
Rural area	0.78 (0.34–1.80)	0.561		
Residence municipality				
Manaus#				
Metropolitan region	1.11 (0.67–1.84)	0.694		
Others	0.70 (0.35–1.41)	0.321		

^#^Reference groups.

### Impact of HIV status on malaria hospitalizations

Six hospitalizations due to *P. vivax* malaria were documented in the HIV-positive group (2.7%) and 40 in the HIV-negative group (4.5%) [RR = 0.58 (0.22–1.32); *P* = 0.216]. No deaths from malaria were reported.

### Impact of CD4+ counts and viral load on malaria incidence and recurrence of *P. vivax* in HIV-positive patients

Among individuals with HIV-positive status, no association was found between CD4+ counts at baseline and the rates of total malaria and *P. vivax* malaria. Individuals carrying a viral load that was < 50 copies/mL at baseline presented a higher risk of malaria [ARR = 3.43 (2.01–5.85); *P* < 0.001] and *P. vivax* malaria [ARR = 3.40 (1.99–5.80); *P* < 0.001] compared to those with a viral load > 100,000 copies/mL, after adjusting by gender, ethnicity, municipality of residence, years of education and zone of residence (rural or urban) (Table 4). CD4 + counts and viral load were not associated with recurrences in *P. vivax* malaria (Table 4).

**Table 4:** Impact of HIV-status and other variables on *P. vivax* recurrences in HIV-positive patients in the state of Amazonas, Brazil, 2012–2020.

Variable	RR (CI95%)	*P*	ARR (CI 95%)^¶^	*P*
CD4 cell counts (/mm^3^)				
< 200#	1	*–*	1	*–*
200–350	1.10 (0.32–3.76)	0.875	1.07 (0.30–3.97)	0.692
350–500	1.78 (0.54–5.85)	0.344	2.01 (0.40–6.37)	0.317
> 500	0.89 (0.26–3.06)	0.852	0.76 (0.22–3.14)	0.890
Viral load (copies/mL)				
> 100,000#	1	–	1	–
10,000–100,000	0.59 (0.12–2.93)	0.519	0.57 (0.12–2.67)	0.479
1000–10,000	1.77 (0.47–6.64)	0.395	2.01 (0.54–7.51)	0.302
50–1000	0.59 (0.07–4.71)	0.619	0.545 (0.08–3.94)	0.548
< 50	0.83 (0.26–2.68)	0.755	0.749 (0.24–2.36)	0.623

^#^Reference groups.

^¶^Adjusted by gender and years of education.

### Impact of HIV status on malaria parasitemia

No significant differences were found in the distribution of parasitemia between HIV-negative and HIV-positive *P. vivax* malaria patients.

## Discussion

The high prevalence of HIV and *P. vivax* malaria in tropical and subtropical regions may increase the simultaneous co-infection by these two pathogens. In this study, HIV-positive status was associated to increased risk of *P. vivax* malaria, as previously observed in Africa with *P. falciparum* malaria^3,13,37^. To the best of our knowledge, there is no information on the effect of HIV infection on *P. vivax* susceptibility, but as occurs in *P. falciparum* malaria^38–40^, abnormalities in the immune response may increase susceptibility to *P. vivax* malaria by compromising naturally acquired immunity^41–43^. A mixed pattern of proinflammatory and regulatory biomarkers involving a robust IL-10 and IL-6 axis is produced in *P. vivax* malaria^43^ and, in recurrent *P. vivax* episodes, an unbalanced CD4^+^/CD8^+^ T-cell ratio was found and is associated with a significant increase in IL-10 levels^44^. Genetic polymorphisms in TLRs play a role in *P. vivax* susceptibility and parasitemia^42^, possibly by activation of IL-6, IFN-γ, IL-10 pathways^45^. From an integrated perspective, mechanisms behind recurrences and subclinical *P. vivax* infections are related to inhibitory receptors, T regulatory cells, and IL‐10, with a poor induction of immunological memory cells and inefficient T effector cells^46^. Since HIV infects CD4 + T cells, a disruption of the components of both the cellular and humoral aspects of the immune response is observed, thus predisposing individuals to many infections^43^.

Evidence from modelling studies^47^ and historical observations suggests that fevers from other systemic parasitic and bacterial infections can activate *P. vivax* hypnozoites^48^. If this is true, opportunistic infections in HIV/AIDS patients could also trigger new episodes of *P. vivax* malaria through relapse. Additionally, reports also suggest that antimalarial treatment failure in *P. falciparum* malaria is more common in HIV-infected patients with low CD4-cell counts compared to those not infected with HIV^49^. In this study, HIV-positive status was associated with a tendency towards protection from recurrences of *P. vivax* malaria. Although some preliminary evidence demonstrated antiplasmodial^50^ and hypnozoiticidal^51^ activities of HIV protease inhibitors, our results point to another explanation for a lower frequency of recurrences in HIV patients, since, overall, *P. vivax* malaria was significantly less common in this group. Moreover, prophylaxis of opportunistic infections using trimethoprim/sulfamethoxazole would possibly minimize the clinical manifestations of recurrences, as seen in *P. falciparum* malaria^52^. As the HIV patients in this study were followed in a tertiary institution for infectious diseases, including malaria, they should have received more information about the need to complete antimalarial treatment, in a more rigorous clinical follow-up to reach a radical cure. It should also be considered that participants with HIV/AIDS may have a lower body weight than those in the HIV-negative group, which could lead to proportionally higher plasma levels of primaquine and its active metabolites, resulting in higher radical cure rates.

In this study, no significant differences were found between HIV-negative and HIV-positive *P. vivax* malaria patients in the distribution of parasitemia and severity. This differs from studies linking HIV to *P. falciparum* parasitemia, which demonstrate that HIV is associated with an increase in parasitemia and severity^53–56^. However, the number of severe cases was very small, which may have limited the statistical power of this comparison.

To the best of our knowledge, this is the only cohort that has been studied to assess the influence of HIV infection on *P. vivax* infections. Regardless of the large number of coinfections, some limitations were imposed by the study design, and these should be stated. HIV patients may be more likely than their HIV negative counterparts to seek treatment for a febrile illness than HIV-negative individuals. HIV patients are more likely to need ongoing outpatient monitoring and therefore will interact with the medical system more frequently.

In accordance with the case reporting procedures, only positive results for malaria are registered in SIVEP-malaria, which prevents to determine the universe of tested individuals, both in HIV positive and controls, potentially generating a substantial detection bias. In the Brazilian Amazon, however, fever is closely associated with malaria by the inhabitants and, in the case of the presence of this sign, testing for malaria is routine in all health units. As a retrospective cohort study, such confounders related to different access to the health system could not be collected. Additionally, as per the Brazilian Ministry of Health and local protocols, the treatment for both diseases is provided free-of-charge and under prescription, which means the diseases are necessarily conditioned to case reporting, and this is expected to minimize underreporting and related bias. Another weakness is regarding the absence of a unique identifier that allows for a deterministic linkage between distinct databases. In order to ensure high accuracy in this process, we used a high throughput algorithm that was previously described^35^ and that has both high sensitivity and specificity when used for probabilistic linkage of public health datasets.

This study hypothesizes that HIV-infected patients are more likely to present *P. vivax* malaria. HIV counselling and testing programs, as well as approaching patients with febrile illnesses for the diagnosis of malaria should be strengthened to improve HIV/AIDS control strategies and the determination of malaria and HIV co-infection to help and articulate prevention and HIV/malaria control. With continuing interest in the elimination of malaria, it is necessary to have a better understanding of HIV-*P. vivax* co-infection, with the goal of reducing the burden of *P. vivax* malaria. Prospective studies are needed to confirm our hypothesis.

## Methods

### Ethics statement

This study was conducted in accordance with the principles of the Declaration of Helsinki and the guidelines of Good Clinical Practice of the International Harmonization Conference. The study was approved by the Ethics Review Board (ERB) of the Fundação de Medicina Tropical Dr. Heitor Vieira Dourado (CAAE: 09,714,619.2.0000.0005). The ERB gave a waiver for informed consent. After database linkages, the final dataset was anonymized before statistical analysis.

### Study site

Out of all malaria cases in Brazil, more than 99% occur in the Brazilian Amazon region, of which about 89% are attributable to *P. vivax* infections^28^. The national HIV prevalence is estimated at 0.4%, with a noticeably higher HIV incidence rate of 34.8/100,000 population in the Brazilian Amazon, compared to the overall national rate of 17.8/100,000^29^.

FMT-HVD is a tertiary, referral healthcare center for infectious diseases in Manaus, state of Amazonas, western Brazilian Amazon and, in its outpatient clinic and emergency department, it receives all patients seeking care for infectious and parasitic diseases or those referred from other health units in the city of Manaus and the surrounding areas. HIV testing is provided after careful health screening for possible epidemiological risk and exposure. Diagnosis and treatment are free of charge. For both diseases, any positive HIV or malaria diagnoses are compulsorily recorded in structured forms available on-line as part of the Brazilian Ministry of Health's notification system. FMT-FVD is responsible for the follow-up and management of ~ 80% of the HIV/AIDS cases in the state of Amazonas.

### Study design and exposure

This is a concurrent, cohort study, based on surveillance data from patients tested for HIV at FMT-HVD and malaria diagnosis data and case recurrences recorded in the National Malaria Epidemiological Surveillance Information System (SIVEP Malaria) between January 2012 and December 2016. In this study, exposure was defined as a confirmed HIV-positive status, according to the national HIV infection diagnosis guideline^30^, as reported on the FMT-HVD Information System (iDoctor).

In short, a rapid diagnostic test (RDT) (*Bio-Manguinhos,* Fiocruz) with 100% sensitivity and 99.8% specificity^31^ was used in mobile testing campaigns and at the FMT-HVD outpatient clinics. For each positive test, blood samples were collected at the FMT-HVD laboratory, and the western blot technique was used to confirm the diagnosis, and the patient was then referred to the HIV ward for consultations, psychological support and ART therapy. All the remaining individuals with HIV-negative test results were included as non-exposed controls. HIV-positive patients were stratified by CD4 cell counts (/mm^3^) and viral load (copies/mL), via data gathered from the FMT-HVD SISCEL (*Laboratory Test Control System*) database. The predictor variables sex, age, education (in years of schooling), self-reported ethnicity, place of residence (rural/urban), and municipality of residence were also used. All HIV-positive patients were treated according to the Brazilian Ministry of Health Guidelines^32^.

### Outcomes

The present study was designed to determine the risk of (i) malaria infection, (ii) *P. vivax* malaria, (iii) *P. falciparum* infection, (iv) *P. vivax* malaria recurrences up to 90, 180 days and 4-years, (v) malaria-associated hospitalizations, (vi) malaria-associated deaths, in a 4-year follow-up, and (vii) parasitemia. Recurrence was defined as the occurrence of at least one additional malaria infection during the follow-up period. Furthermore, the study aimed to determine any association between age, sex or parasitemia as a potential additional risk factor for malaria reinfection. In Brazil, thick blood smears (TBS) are routinely performed for the diagnosis of malaria, and are prepared according to the Walker technique^33^ and then evaluated by a local microscopist. The results of peripheral parasitemia are given using the following semi-quantitative system: < 1/2 + (< 200 parasites/mm^3^); 1/2 + (200–300 parasites/mm^3^); 1 + (301–500 parasites/mm^3^); 2 + (501–10,000 parasites/mm^3^); 3 + (10,001–100,000 parasites/mm^3^); and 4 + (> 100,000 parasites/mm^3^). All positive slides and 10% of negative slides are routinely reviewed at a referral unit with experienced microscopists. In case of divergence, the reviewed result is updated on the on-line system. Only participants with a positive TBS for *Plasmodium* spp. are treated according to the Brazilian Anti-Malarial Treatment Guidelines^34^. The malaria patients' data are entered into the SIVEP Malaria (*National Malaria Surveillance*) database. Data on malaria-associated hospitalizations and deaths were extracted from HIS (*Hospitalization Information System*) and MIS (*Mortality Information System*) databases, respectively.

### Data processing and record linkage strategy

Duplicated HIV tests were excluded from the iDoctor database (HIV-status database; 2012–2016 period) and, in the case of individuals that had had more than one HIV test in the period, the first positive result was considered. Patients exposed to disease (HIV-positive) and the controls (HIV-negative) were included in the study according to the day of confirmation of HIV-status. Patients entered and left the study at different points in time. The HIV-status database and the SIVEP-malaria database were both deduplicated and further the record linkage was performed (Malaria database; 2012–2020) of Tucuxi-BLAST software, which uses the DNA-encoded approach^35^ and was installed on a Linux Workstation, Intel Core i7-8700, with 32 GB of RAM and 12 physical processing cores. In the absence of a unique identifier, Tucuxi-BLAST used the patient's name, date of birth and mother's name. Malaria episodes reported before the participants' entries were then excluded from the generated database. All remaining matches were double-checked and false matches were also excluded. This inspection included visual confirmation of homonyms, possible siblings (twins), and duplicity. Finally, the list of malaria cases obtained was linked to the HIS (*Hospitalization Information System*) and MIS (*Mortality Information System*) databases to investigate these outcomes. We obtained a final selection of pairs identified as likely to be from the same patients by automatic verification, applying a probability threshold (probability > 0.7) for all linkages. A final identification (ID) was created for all included participants.

### Statistical analysis

Descriptive statistics were used for demographic variables. Student's t test was used to compare means while Fisher's exact or the Chi-squared (X^2^) test were used to compare proportions, as appropriate. Crude relative risk (RR) with its respective 95% confidence interval (95% CI) was determined in a univariate analysis. Logistic regression was used for the multivariate analyses and the adjusted RR (ARR) with 95% CI were also estimated. A log binomial multivariate generalized linear regression was performed using an automated backward and forward stepwise estimation. All variables that were associated with dependent variables at a significance level of *P* < 0.200 in the univariate analysis were included in the multivariate analysis. Statistical significance was considered when *P* < 0.05 in the Hosmer–Lemeshow goodness-of-fit test. Kaplan–Meier survival estimates were used to compare recurrences over follow-up time. Additionally, a survival analysis for recurrent events was performed^36^. A two-tailed *P* < 0.05 was considered significant. The statistical analyses were carried out using R software (version 4.1.0), R Studio (version 1.4.17), and Stata v.13.0 (Stata Corp. LP, College Station, TX).

## Supplementary Information

Supplementary Information.

## Data Availability

All data generated or analysed during this study are included in this published article [and its supplementary information files].
